# Comparative study of dual energy CT iodine imaging and standardized concentrations before and after chemoradiotherapy for esophageal cancer

**DOI:** 10.1186/s12885-018-5058-2

**Published:** 2018-11-16

**Authors:** Xiaomin Ge, Jingping Yu, Zhongling Wang, Yiqun Xu, Changjie Pan, Lu Jiang, Yanling Yang, Kai Yuan, Wei Liu

**Affiliations:** 1 Department of Radiology, Changzhou Second People’s Hospital Affiliated to Nanjing Medical University, No. 29 Xinglong Road, Tianning District, Changzhou, Jiangsu China; 2 Department of Radiotherapy, Changzhou Second People’s Hospital Affiliated to Nanjing Medical University, Changzhou, 213003 China; 3 Department of Radiology, Shanghai First People’s Hospital Affiliated to Shanghai Jiao Tong University, Shanghai, 200080 China; 4 Thoracic Surgery Department, Changzhou Second People’s Hospital Affiliated to Nanjing Medical University, Changzhou, 213003 China

**Keywords:** Dual energy CT, Iodine imaging, Esophageal cancer, Chemoradiotherapy

## Abstract

**Background:**

To compare dual energy CT iodine imaging and standardized iodine concentration before and after chemoradiotherapy (CRT) for esophageal cancer and evaluate the efficacy of CRT for EC by examining DECT iodine maps and standard CT values.

**Methods:**

The clinical data of 45 patients confirmed by pathology with newly diagnosed esophageal cancer who underwent concurrent CRT from February 2012 to January 2017 in our department of radiology were collected. All patients underwent dual-source dual-energy CT (DECT) before and after CRT. Normalized iodine concentration (NIC) and normalized CT (NCT) corresponding to the overall cancer lesion and its maximum cross-sectional area were observed and compared. Additionally, 30 healthy individuals were compared as control group. After treatment, the patients were divided into two groups according to RECIST1.1: treatment effective group and ineffective group.

**Results:**

There were 33 patients (CR 9, PR 24) in the effective group and 12 patients (SD 12, PD 0) in the ineffective group. There was no significant difference in the NIC-A, NIC-V, NCT-A and NCT-A indexes between the effective group (B group) and the ineffective group (C group) before treatment (*P* > 0.05). After the treatment, the above-mentioned indexes in the effective group of patients were significantly lower than before treatment, and compared with the ineffective group, the NIC-A, NIC-V, NCT-A and NCT-V values of the effective group were significantly lower than those of ineffective group (*P* < 0.05). After treatment, the NIC-V and NCT-V in the ineffective group were lower than before treatment, and the difference was statistically significant (*P* < 0.05). However, their NIC-A and NCT-A were not statistically different from those before treatment (*P* > 0.05).

**Conclusion:**

Using DECT iodine map, the changes of NIC and NIC before and after CRT in patients with esophageal cancer can evaluate the effect of CRT, and does not increase the radiation dose, so it is suitable for clinical use.

## Background

Esophageal cancer (EC) is considered a serious malignancy with respect to prognosis and mortality rate. Accounting for more than 509,000 deaths worldwide in 2018 [1]. It is the eighth most common cancer and the sixth most common cause of cancer-related deaths worldwide with developing nations making up more than 80% of total cases and deaths [2].

Early symptoms of EC is not obvious, the pathological changes are not obvious to the mucosal surface. Most patients at the time of diagnosis have lost their chance of surgery, and the effect of surgical treatment on advanced EC patients is reduced. Chemoradiotherapy (CRT) has become one of the main treatments for advanced EC [3].

CT plain scan through the thickness of esophageal lesions to determine the treatment effect shows not only low contrast, but it is also difficult to accurately present the relationship between the internal lesion and the surrounding tissue, thus the judgment of clinical efficacy is lower [4]. With the continuous development of CT technology, dual-source dual-energy CT (DECT) is widely used clinically, it can obtain mixed energy images and fused images through data processing in many different forms of images, synthesize monoenergetic images at different energies, such as iodine maps, virtual noncontrast images and single-energy images, which has a certain meaning to changes in the display of lesions before and after treatment [5]. It was shown that enhanced CT iodine map in evaluating the effectiveness of treatment of tumors had a good value [6]. However, there the efficacy of DECT in the evaluation of CRT for EC was rarely investigated. Whether DECT was better than conventional CT scan in the evaluation of CRT for EC is still unknown. Therefore, this study was to evaluate the efficacy of CRT for EC by examining DECT iodine maps and standard CT values, designed to effectively assess the patient’s disease progression and guide the next step of treatment.

## Method

### General information

We collected 45 patients with EC from our department of radiology from February 2012 to December 2017. There were 26 males and 19 females with a mean age range of 38 to 76 years, with an average of age (61.35 ± 5.84) years old. The patients were divided into: group A (*n* = 45), before CRT; group B (*n* = 33), remission after CRT; group C (*n* = 12) without remission after CRT, and Group D (*n* = 30), healthy controls including 15 males and 15 females, the age range of 40–75 years, average age of 61.35 ± 5.84 years.

Patients inclusion criteria: 1. Single lesions and complete resection; 2. Postoperative pathology confirmed; 3. Enhanced scan found tumor; 4. Exclude other combined diseases such as esophagitis, esophageal tuberculosis; 5. The barium swallow angiography or endoscopy to identify the lesion; 6. Without any systematic treatment. Exclusion criteria: 1. Clinical data missing or incomplete; 2. Contraindications to the use of iodine contrast agents; 3. Other esophageal diseases; 4. Patients cannot accept iodine enhanced CT; 5. Radiotherapy or chemotherapy (CMT) treatment history.

### Method

#### Treatment methods

##### Radiotherapy

Radiotherapy using 6MV X-ray irradiation, intensity-modulated radiation therapy (IMRT), the tumor target area containing tumor, thickened esophageal wall (> 5 mm), lymph nodes (short axis diameter of 5 mm or more) of cardio phrenic angle, Para esophageal, and tracheoesophageal groove and metastatic mediastinal lymph nodes (more than 1 cm). The clinical target area was 5–8 mm anterior, posterior and lateral margins and 3 cm cranial and caudal margins to the tumor. Patients received 1.8 Gy per day, 5 times per week, a total dose of 45.0–50.4 Gy. CMT regimen: The TP regimen (paclitaxel + cisplatin / carboplatin) was used to standardize CMT for 2 cycles. Each patient underwent DECT before treatment and after treatment for 4 weeks.

#### CT inspection method

All patients underwent fasting for 8-12 h prior to CT scanning and utilized the supine position for routine scan positioning image. Scanning range: from the thorax entrance to the entrance to the cardia. Before initiating contrast-enhanced scanning, a high-pressure syringe was used to inject 70 ml of non-ionic contrast agent iohexol (300 mg / ml) 70 ml through the elbow vein at an injection rate of 3 ml/s. All patients were examined using a dual-source CT system (Definition, Siemens, Forchheim, Germany) in dual-energy mode. The dual-energy scanning mode: Tube A of the dual-source CT system was operated at 180 mAs/rot at 100 kV and the tube B at 90 mAs/rot at 140 kV. Dedicated automated real-time attenuation-based tube current modulation software (CARE Dose 4D; Siemens) was activated. After injection of contrast agent, patients were subjected to double helical scanning at hepatic arterial phase (HAP) and portal venous phase (PVP) 25 s and 60 s while holding their breath. The image acquisition layer has a thickness of 0.5 mm and a helical pitch of 1.2, the rotation time was 0.5 s. We reconstructed an iodine map and composite images at 120 kV (120 kV images) using raw data with scan parameters of 50% of 100 Kv and 50% of 140 kV.

### Image post-processing and parameter measurement

For all individuals, DECT data were transferred to a workstation (MMWP, Germany) for further analysis. The dual-energy datasets were post-processed using clinically available dedicated software. The Liver VNC application class (Syngo, Dual Energy, Siemens Medical Solutions, Erlangen, Germany) was used to reconstruct transverse VNC images without changing slice thickness or reconstruction intervals. The images were assessed by two experienced radiologists who reached consensus on the diagnosis. The axial, sagittal and coronal images were observed. The maximum diameter of lesions was measured at the maximal level of the lesion. The region of interest (ROI) with area of 0.6 ~ 1.0 mm^2^ was measured 3 times to obtain the average. At this time, care should be taken to avoid tumor margins and necrotic areas. The ROI size / slice level measured in the arterial and venous phases are approximately the same. The ROI of group D was a randomly selected esophageal normal mucosa with an area of 0.2–0.4 mm^2^, and measurements were averaged 3 times. To reduce individual differences, iodine concentration (IC) and CT values were normalized. Normalized iodine concentration (NIC) and normalized CT (NCT) values were calculated according to following formulas:

$$ \mathrm{NIC}-\mathrm{A}=\mathrm{HAP}-\mathrm{ROI}\;\mathrm{iodine}\kern0.17em \mathrm{concentration}/\mathrm{simultaneous}\ \mathrm{aortic}\ \mathrm{phase}\ \mathrm{iodine}\ \mathrm{concentration} $$

$$ \mathrm{NIC}-\mathrm{V}=\mathrm{PVP}-\mathrm{ROI}\ \mathrm{iodine}\ \mathrm{concentration}/\mathrm{simultaneous}\ \mathrm{aortic}\ \mathrm{phase}\ \mathrm{iodine}\ \mathrm{concentration} $$

$$ \mathrm{NCT}-\mathrm{A}=\mathrm{HAP}-\mathrm{ROI}\ \mathrm{CT}\ \mathrm{value}/\mathrm{simultaneous}\ \mathrm{aortic}\ \mathrm{phase}\ \mathrm{CT}\ \mathrm{value} $$

$$ \mathrm{NCT}-\mathrm{V}=\mathrm{PVP}-\mathrm{ROI}\ \mathrm{CT}\ \mathrm{value}/\mathrm{simultaneous}\ \mathrm{aortic}\ \mathrm{phase}\ \mathrm{CT}\ \mathrm{value} $$

### Assessment of cancer treatment according to RECIST 1.1 [7]

Observed indicators are morphology and size of the lesion, changes of lymph nodes around the lesion, and tumor target lesion maximum diameter and the relative baseline level ratio before and after CRT. Complete response (CR): After treatment, the target lesion disappeared at the tumor site, the short diameter of all lymph nodes (including target nodules and non-target nodules) was reduced less than 10 mm from before treatment; Partial response (PR): At least a 30% decrease in the sum of diameters of target lesions, taking as reference the baseline sum diameters, in the absence of new lesions or unequivocal progression of non-index lesions; Progressive disease (PD): At least a 20% increase in the sum of diameters of target lesions, taking as reference the smallest sum on study. The sum must also demonstrate an absolute increase of at least 5 mm, or one or more new lesions appeared. Stable disease (SD): Neither sufficient shrinkage to qualify for PR nor sufficient increase to qualify for PD, taking as reference the smallest sum diameters, in absence of new lesions or unequivocal progression of non-index lesions. In effective group, CR or PR was evaluated, while in ineffective group SD or PD was evaluated.

### Statistical analysis

Statistical analysis was performed for parameters using SPSS 22.0. The results were presented as mean ± standard deviation (SD). Among the four groups, arterial and venous NIC and NCT values were compared using one-way ANOVA one-way ANOVA followed by Newman-Keus test. The enumeration data were analyzed with the chi-square test. *P* < 0.05 was considered statistically significant.

## Results

### Patients’ data

Among the 45 patients, the distribution of the tumor site was: 4 cases of cervical segment, 13 cases of upper thoracic segment, 19 cases of middle thoracic segment and 9 cases of lower thoracic segment. The clinical stage was stage III in 31 cases and stage IV in 14 cases, according to the Union for International Cancer Control (UICC), TNM Classification of Malignant Tumors, 8th edition. The pathological type was squamous cell carcinoma in 32 cases, adenocarcinoma in 8 cases and small cell carcinoma in 5 cases. After 2 cycles of CMT, 33 patients (CR 9 cases, PR 24 cases) was classified as group B, 12 patients (SD 12 cases, PD 0 cases) as group C.

### Comparing NIC and NCT value of HAP and PVP in four groups

The NIC and NCT value of HAP and PVP between group A and group B and group D were significantly different (*P* < 0.05). The NIC-A, NIC-V, NCT-A and NCT-A in esophageal mucosal arteries in group A and C were significantly different from those in group D (*P* < 0.05). There was no significant difference in NIC-A, NIC-V, NCT-A and NCT-A between group A and group C (*P* > 0.05). The NIC-A, NIC-V, NCT-A and NCT-A in esophageal mucosal arteries in group B were significantly different from those in group C (*P* < 0.05). There were no significant differences in the NIC-A, NIC-V, NCT-A and NCT-A values of groups B and D (*P* > 0.05, Table 1).

**Table 1:** Comparison of NIC-A, NIC-V, NCT-A and NCT-V (mean ± SD)

Groups	NIC-A	NCT-A	NIC-V	NCT-V
Group A (*n* = 45)	0.28 ± 0.04^bd^	0.33 ± 0.06^bd^	0.54 ± 0.06^bd^	0.57 ± 0.07^bd^
Group B (*n* = 33)	0.23 ± 0.05^ac^	0.27 ± 0.04^ac^	0.47 ± 0.06^ac^	0.50 ± 0.04^ac^
Group C (*n* = 12)	0.29 ± 0.06^bd^	0.31 ± 0.04^bd^	0.51 ± 0.06^bd^	0.54 ± 0.05^bd^
Group D (*n* = 30)	0.24 ± 0.05^ac^	0.28 ± 0.07^ac^	0.48 ± 0.07^ac^	0.49 ± 0.06^ac^
F value	9.11	6.67	13.36	15.86
*P* value	< 0.01	< 0.01	< 0.01	< 0.01

^a^*P* < 0.05 compared with group A

^b^*P* < 0.05 compared with group B

^c^*P* < 0.05 compared with group C

^d^*P* < 0.05 compared with group D

### Comparison of parameters before and after treatment in group B and C

**Fig. 1:**
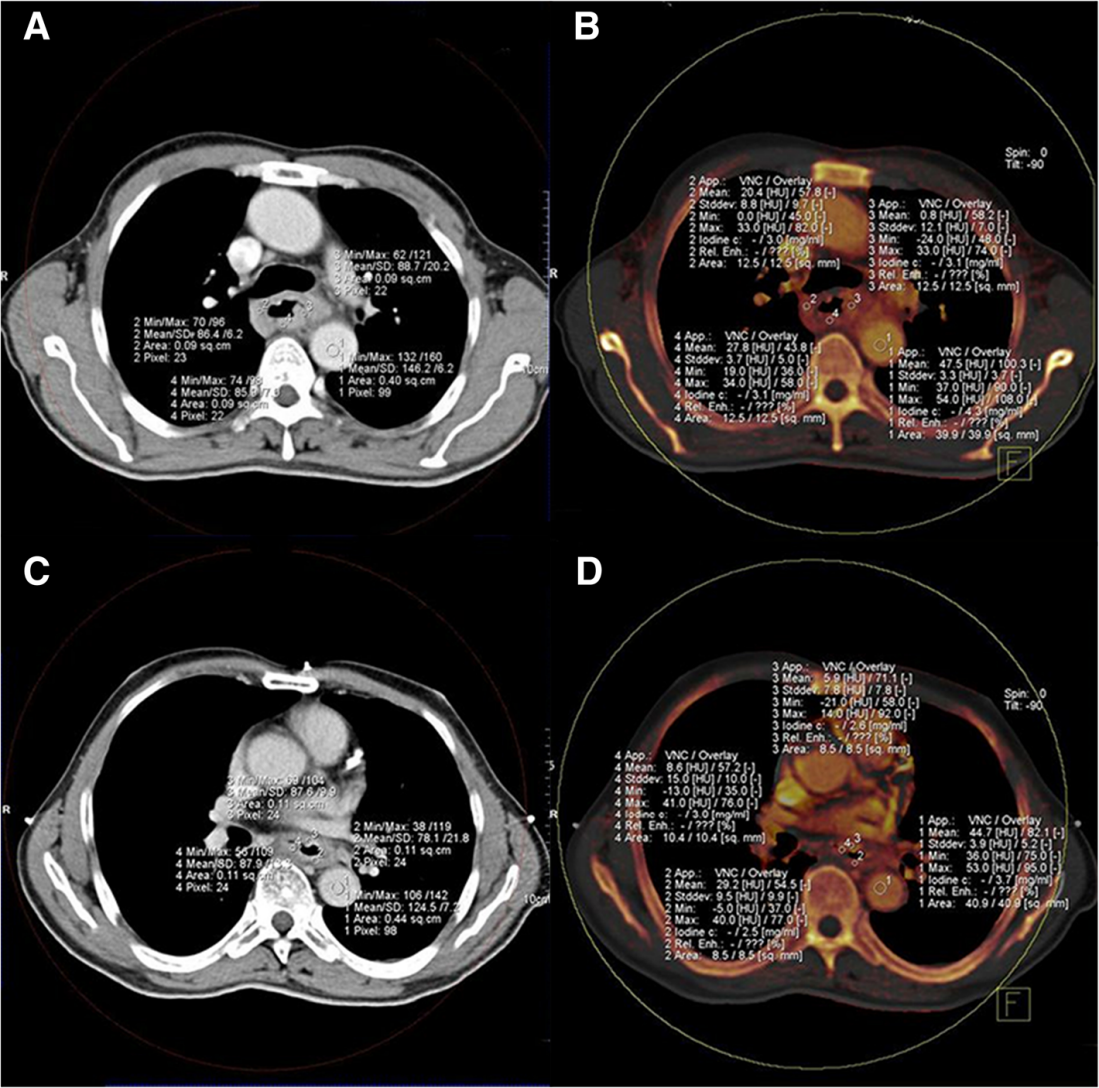
CT images of esophageal cancer before and after treatment. **a** The mucosal and aortic CT value of esophageal cancer before treatment; **b** The mucosal and aortic iodine values of esophageal cancer before treatment; **c** The mucosal and aortic CT value of esophageal cancer after treatment; **d** The mucosal and aortic iodine values of esophageal cancer after treatment

There was no significant difference in the NIC-A, NIC-V, NCT-A and NCT-A indexes between the effective group (B group) and the ineffective group (C group) before treatment (*P* > 0.05). After the treatment, the above-mentioned indexes in the effective group of patients were significantly lower than before treatment, as compared with the ineffective group, their NIC-A, NIC-V, NCT-A and NCT-V were significantly lower than the ineffective group, the differences were statistically significant (*P* < 0.05). After treatment, the NIC-V and NCT-V in the ineffective group were lower than before treatment, and the difference was statistically significant (*P* < 0.05). However, their NIC-A and NCT-A were not statistically different from those before treatment (*P* < 0.05, Table 2, Fig. 1a, b, c and d).

**Table 2:** Comparison of NIC-A, NIC-V, NCT-A and NCT-V before and after treatment in group B and C (mean ± SD)

Indexes	Group B (*n* = 33)		Group C (*n* = 12)	
	Before CRT	After CRT	Before CRT	After CRT
NIC_-A_	0.28 ± 0.05	0.23 ± 0.05^*#^	0.28 ± 0.03	0.29 ± 0.06
NIC_-V_	0.54 ± 0.06	0.49 ± 0.06^*#^	0.56 ± 0.06	0.51 ± 0.06^*^
NCT_-A_	0.34 ± 0.06	0.27 ± 0.04^*#^	0.32 ± 0.04	0.31 ± 0.04
NCT_-V_	0.57 ± 0.8	0.5 ± 0.04^*#^	0.59 ± 0.04	0.54 ± 0.05^*^

*P < 0.05 compared with before treatment

^#^*P* < 0.05 compared with the ineffective group

### Measurement for effective radiation dose

The average radiation dose (2.36 ± 1.67 millisieverts cm) in DECT was significantly different from the 64-slice CT mode (5.65 ± 1.81 millisieverts cm) (*P* < 0.05).

## Discussion

As a common malignant tumor, EC has rich blood vessels and high metabolism. High-energy radiation is used to irradiate the EC cells by using radiotherapy equipment. High temperature can directly cause cells damage or damage the intracellular structure, thereby leading to cell apoptosis [8]. Through direct destruction of intracellular hydrocarbons generated by oxidation and formation of free radicals further interfered with DNA synthesis, it could inhibit tumor cell proliferation. Adjuvant CMT drugs can directly damage the tumor cell DNA or tumor cell membrane components. In this study, the selection of platinum drugs also played the role in inhibiting RNA and protein synthesis, clinical reports showed that cisplatin has a good anti-cancer effect in the treatment of EC [9–11]. Studies have shown that after cancer patients were treated with standard radiotherapy and CMT, the target tumor cells showed apoptosis, the process of cell proliferation was blocked and the number of cells was decreased and the target lesion showed artery necrosis, thus the entire tumor showed necrosis, reduced or disappeared [12].

Iodine map is an important analytical parameter in DECT imaging, which can directly reflect the difference of IC in the tumor and indirectly reflect the blood supply in the lesion. Through the post-processing, the IC in the lesion can be quantitatively measured. Quantitative analysis with IC not only can improve the diagnostic accuracy, but also specify the target lesions in patients with EC [13, 14]; the rate of change of IC is related to its pathological tumor grade after CMT [15]. DECT using two types of tube voltage has enabled quantification of the iodine-related attenuation (IRA) of iodinated contrast material in tumors after intravenous injection, without the need for an additional non-contrast CT scan. Recent study has shown that three-dimensional iodine-related attenuation (3D-IRA) measured by DECT is correlated with degree of differentiation and locoregional invasion in primary lung cancers [16]. In recent years, a study found that the higher the degree of differentiation of tumor tissue and pathological staging, the more dense the blood vessel density, and the degree of enhancement of the lesion has a close relationship with the vascular density, body blood vessels on the iodine uptake rate is different, the iodine in the iodine map shows iodine concentration (IC), iodine value, CT value and other parameters are also different [17].

Our results showed that the NIC-A, NIC-V, NCT-A and NCT-V after treatment in the effective group were significantly lower than before treatment, and the NIC-V and NCT-V in the ineffective group were lower than those before treatment. After treatment, NIC-A, NIC-V, NCT-A, and NCT-A in esophageal cancer were all lower than those before treatment, indicating that CRT treatment led to reduced blood supply of EC, inhibited tumor cell proliferation, decreased tumor cells number, and thus reflected by the reduced tumor cells iodine intake, which is consistent with the previous study [18]. In addition, CMT drugs can exert toxic effects on cells and destroy vascular endothelial activity, induce necrosis of tumor lesions, mucosal congestion and edema, and inflammatory reactions, etc. These can effectively inhibit the proliferation of tumor blood vessels and reduce the blood supply in the cancer area, then reduce the uptake of iodine by tumor cells. Vascular IC can reflect the efficacy of CMT drugs within the tissue [16, 19–26].

The NIC-A and NCT-A values of the patients in the ineffective group were not significantly different from those before the treatment. This may be because after CRT, the absorption of iodine in the HAP was relatively low, so there was no significant difference between the two groups of values. On the other hand the amount of iodine in the PVP was absorbed to a certain extent, CRT still has some effects. Our data showed that NIC-V and NCT-V were both higher than NIC-A and NCT-A, respectively. Chen et al. [27] suggested that the bi-phasic IC had a positive linear correlation with micro-vessel density (MVD). PVP IC reflected the angiogenesis in relatively earlier and well-differentiated advanced gastric cancer, while HAP IC reflected this in further advanced and poorly differentiated gastric cancer. Spectral CT with quantitative IC value offers a new choice to evaluate the angiogenesis of gastric cancer noninvasively.

In this study, there was no significant difference in NIC-A, NIC-V, NCT-A, NCT-V between the effective and ineffective groups before treatment. After treatment, the above indicators of effective group were significantly lower than before treatment. Compared with the ineffective group, the NIC-A, NIC-V, NCT-A and NCT-V of effective group were significantly lower, confirming the esophageal IC can functionally assess the efficacy of CRT. IC is complementary to traditional morphological assessment and it shows more prognostic information.

In terms of radiation dose, we compared with the conventional CT scan mode and found no significant difference in mean radiation dose between DECT and conventional CT scan, indicating that DECT did not cause additional radiation damage, it was safe and effective, and was suitable for efficacy evaluation.

## Conclusion

DECT iodine imaging scan performed before and after CRT of EC can be used as a traditional morphological indicator to evaluate the prognosis. DECT can provide valuable information for the treatment and prognosis of patients. Compared with conventional CT scan, DECT does not increase radiation dose, which is suitable for clinical utilization.
